# *Angiostoma norvegicum* n. sp. (Nematoda: Angiostomatidae) a parasite of arionid slugs in Norway

**DOI:** 10.1007/s11230-016-9674-4

**Published:** 2017-01-06

**Authors:** Jenna L. Ross, Solveig Haukeland, Bjørn A. Hatteland, Elena S. Ivanova

**Affiliations:** 1 Department of Conservation Ecology and Entomology, Faculty of AgriSciences, Stellenbosch University, Private Bag X1, Matieland, 7602 South Africa; 2 Institute of Biological and Environmental Sciences, University of Aberdeen, Aberdeen, AB24 3UU UK; 3 ICIPE, African Insect Science for Food and Health, P.O. Box 30772-00100, Nairobi, Kenya; 4 Norwegian Institute of Bioeconomy Research (NIBIO), Pb 115, 1431 Ås, Norway; 5 Department of Biology, University of Bergen, P.O. Box 7800, 5020 Bergen, Norway; 6 Norwegian Institute of Bioeconomy Research (NIBIO), Plant Health and Biotechnology, NIBIO Ullensvang, 5781 Lofthus, Norway; 7 Centre of Parasitology, A.N. Severtsov Institute of Ecology and Evolution, Russian Academy of Science, Leninskii Prospect 33, 119071 Moscow, Russia

## Abstract

*Angiostoma norvegicum* n. sp. (Angiostomatidae) is described from the oesophagus, crop and the buccal mass of five species of slugs of the family Arionidae, *Arion vulgaris* (Moquin-Tandon), *Arion ater* (L.), *Arion fasciatus* (Nilsson), *Arion fuscus* (Müller) and *Arion rufus/Arion ater* hybrid), collected throughout Norway. *Angiostoma norvegicum* n. sp. was found parasitising arionids at seven of the 30 sample sites examined (23.3%), and 9.9% of all *Arion* spp. were infected with this nematode. The new species is characterised by its large size (4.0–8.6 mm long) and in having: lateral alae; 6 + 6 papillae at the cephalic end; a large circular mouth aperture; a spacious stoma; a pharyngeal basal bulb without valvular apparatus; an excretory pore near the base of bulb; a distal part of posterior ovary always outstretched; an anterior ovary distally nearly always outstretched; a vulva situated anterior to mid-body; long, nearly straight spicules and a small gubernaculum; three circumcloacal papillae and caudal genital papillae (GP) arranged in a pattern 1+2/3+3 with GP 5 and GP 8 opened on dorsal side of narrow bursa not reaching tail tip; short conical tails in both sexes with tips supplied by 4 short, unequal denticles. Morphologically, *A. norvegicum* n. sp. is similar to *Angiostoma limacis* Dujardin, 1845, which diagnostic characteristics are given based on examination of specimens from Norway and the UK. Conversely, the phylogenetic analyses based on D2D3 large subunit (LSU) rRNA gene sequences performed in the present study did not support the morphological affinity of these two species. Phylogenetic analyses demonstrated that although *Angiostoma* spp. cluster together, *A. norvegicum* n. sp. forms a tight monophyletic clade with the milacid nematode parasites *Angiostoma margaretae* Ross, Malan & Ivanova, 2011 and *Angiostoma milacis* Ivanova & Wilson, 2009.

## Introduction

The association between nematodes and terrestrial molluscs is poorly understood; however their host specificity, diversity of specialisations and mechanisms of infection, indicate that their affiliation is both ancient and widespread (Morand et al., 2004). International surveys conducted in Europe, North America, Australasia and Africa (Ross et al., 2016), indicate that there are eight families of nematodes that use terrestrial molluscs as definitive hosts, i.e. the Agfidae Dougherty, 1955, Alaninematidae Théodoridés, 1957, Alloionematidae Chitwood & McIntosh, 1934, Angiostomatidae Blanchard, 1895, Cosmocercidae Railliet, 1916, Diplogastridae Micoletzky, 1922, Mermithidae Braun, 1883 and Rhabditidae Örley, 1880 (see Pieterse et al., 2016; Ross et al., 2016).

The family Angiostomatidae is comprised of two genera: *Angiostoma* Dujardin, 1845, which contains seventeen species (Ross et al., 2011), and *Aulacnema* Pham Van Luc, Spiridonov & Wilson, 2005, which is presently monotypic (Pham Van Luc et al., 2005). Thirteen species of *Angiostoma* are described from molluscan hosts (Dujardin, 1845; Mengert, 1953; Morand, 1986, 1988, 1992; Morand & Spiridonov, 1989; Korol & Spiridonov, 1991; Ivanova & Wilson, 2009; Ivanova & Spiridonov, 2010; Pham Van Luc et al., 2005; Ross et al., 2011) with eight of these species belonging to mainland Europe and the UK (Ivanova et al., 2013). The remaining *Angiostoma* spp. are associated with the intestine and bronchi of amphibian and reptile hosts (Falcón-Ordaz et al., 2008). During a survey of nematodes associated with terrestrial slugs in Norway (Ross et al., 2016), a new species of *Angiostoma* was found together with *Phasmarhabditis hermaphrodita* (Schneider, 1859) Andrássy, 1983, *Agfa flexilis* (Dujardin, 1845), *Alloionema appendiculatum* Schneider, 1859 and another species of the same genus, *Angiostoma limacis* Dujardin, 1845. The new species is described and illustrated below. To demonstrate fine differences between *Angiostoma norvegicum* n. sp. and the morphologically closest *A. limacis*, original illustrations of certain morphological structures of *A. limacis* are also provided.

## Materials and methods

*Slug collection*

*Arion* spp. were collected from 30 sample sites in Norway. Habitats included roadside verges, domestic gardens and agricultural land. Localities were found in cooperation with local advisory services and coordinates were recorded. Slugs were collected during late August, September and October 2011, and identified through morphological examination, dissection of genitalia and analysis of mitochondrial DNA (Ross et al., 2016). Specimens of *A. limacis* used for illustrations of details of diagnostic value were collected from *Arion distinctus* Mabille in Scotland by E. Ivanova in 2006–2007.

*Morphological analysis*

Nematodes were washed with Ringer’s solution from the dissected host into a watch glass and then picked with a needle. Depending on the intensity of infection, between one and four nematodes collected from each species of *Arion* was stored in 70% ethanol for DNA extraction. The remainder of nematodes were fixed by adding hot 4–5% formaldehyde for morphological studies. Measurements and drawings were taken from formaldehyde-fixed nematodes mounted on permanent slides after processing to glycerine following Seinhorst (1959). All measurements are in micrometres, and presented in the text as the range followed by the mean in parentheses. The following abbreviations were used: L, body length; V%, distance from anterior extremity to vulva as a percentage of body length a, b, c, indices of De Man. Genital papillae formula is given as proposed by Sudhaus & Fürst von Lieven (2003). For light microscopy, compound microscopes Zeiss Jenaval and Nikon Eclipse E200 with drawing attachment were used. Illustrations were finalised with WACOM Intuos A4 USB drawing tablet and Adobe Illustrator CS5 following Coleman (2003). For scanning electron microscopy (SEM), nematodes were re-hydrated after formaldehyde, dehydrated in a graded ethanol series, critical-point dried using a HCP-2 HITACHI dryer, mounted on aluminium stubs and coated with gold in a BIO-RAD SC502 sputter coater. Specimens were studied in a JCM-6380 LA SEM and CamScan S2 (Cambridge Instruments, UK).

*Molecular analysis*

Ethanol-preserved nematodes were transferred to a Chelex/Proteinase K mix for DNA extraction (Ross et al., 2010) followed by polymerase chain reaction of the small subunit (SSU) rRNA gene (Ross et al., 2010), D2D3 large subunit (LSU) rRNA gene (Nguyen, 2007; Ivanova & Spiridonov, 2010) and the mitochondrial cytochrome *c* oxidase subunit I (*cox*1) gene (Kanzaki & Futai, 2002). Sequences were assembled using CLC Genomics Workbench 7.6.4 (https://www.qiagenbioinformatics.com/) and submitted to the GenBank database, at the National Centre for Biotechnology Information (http://www.ncbi.nlm.nih.gov/).

*Phylogenetic analysis*

The phylogenetic relationships among *A. norvegicum* n. sp. and several other *Angiostoma* spp. was determined using the D2D3 LSU rRNA gene sequences. Reference sequences downloaded from the NCBI databases included: *Angiostoma glandicola* Ivanova & Spiridonov, 2010 (GQ167724), *A. limacis* (GQ167725), *A. dentiferum* (Mengert, 1953) (GQ167726), *Angiostoma milacis* Ivanova & Wilson, 2009 (FJ949063), *Angiostoma margaretae* Ross, Malan & Ivanova, 2011 (KU712562), along with *Caenorhabditis remanei* (Sudhaus, 1974) (AY602174), *Rhabditella axei* (Cobbold, 1884) (AY602177) and *Oscheius myriophilus* (Poinar, 1986) (AY602176) which were used as the outgroup (Ivanova & Spiridonov, 2010). A total of nine nematode D2D3 LSU rRNA gene sequences were compiled and aligned manually using BioEdit Sequence Alignment Editor (Hall, 1999). Regions of ambiguous alignment were removed leaving 496 aligned characters for analysis. Phylogenetic analyses were performed on unambiguously aligned positions using maximum likelihood (ML), distance and maximum parsimony (MP) analyses, using the software packages PHYML (Guindon & Gascuel, 2003) and PHYLIP (Felsenstein, 2007). Sequence alignments were evaluated using Modeltest, and the general-time reversible (GTR) model was employed along with among-site rate heterogeneity which was modelled based on an eight-category gamma correction with a fraction of invariant sites calculated from ML analysis. Bootstrap support was calculated based on 1,000 replicates. Bootstrap values above 65% were considered.

## Results

General data on the occurrence of *A. norvegicum* n. sp. and other slug-parasitic nematodes in Norway were given in Ross et al. (2016) where the new species was designated as *Angiostoma* sp. A total of 322 *Arion* spp. were collected from 30 sample sites around Norway. *Angiostoma norvegicum* n. sp. was found parasitising five out of seven species of Arionidae at 23.3% of sample sites (Table 1). Of all *Arion* spp. collected, 9.9% were infected with *A. norvegicum* n. sp., which was isolated from the oesophagus, crop and buccal mass of slug hosts. The number of *A. norvegicum* n. sp. recovered from *Arion* spp. varied from 1 to 56 nematodes, with a sex ratio of approximately 2 females: 1 male.

**Table 1:** Prevalence (P in %) and mean intensity (MI) of *Angiostoma norvegicum* n. sp. from arionid slugs in Norway

Host species	n	P (%)	MI
*Arion ater* (L.)	33	12.1	28.0
*Arion circumscriptus* Johnston	4	–	–
*Arion distinctus* Mabille	5	–	–
*Arion fasciatus* (Nilsson)	1	100	4.0
*Arion fuscus* (Müller)	70	1.4	2.0
*Arion ater/Arion rufus* hybrid	6	16.7	14.0
*Arion vulgaris* (Moquin-Tandon)	204	12.3	28.1

*Abbreviation*: n, number of slugs examined

***Angiostoma norvegicum*** **n. sp.**

*Type-host*: *Arion vulgaris* (Moquin-Tandon, 1855).

*Other hosts*: *Arion ater* (Linnaeus), *A. fasciatus* (Nilsson), *A. fuscus* (Müller) and *A. rufus/A. ater* hybrid.

*Type-locality*: Bolsøya (62°43′32″N, 07°18′′01″E), Norway (collected by Haukeland and Ross in September, 2011).

*Other localities*: See Table 2 for details.

**Table 2:** Sample sites in Norway with *Arion* spp. infected with *Angiostoma norvegicum* n. sp.

Location	Coordinates	Habitat	Host species
Bjugn	63°50′35″N, 09°51′27″E	Domestic garden	*Arion vulgaris*
Sandvika; Bolsøya	62°43′36″N, 07°18′44″E	Roadside verge	*Arion ater*
Åsane	60°27′49″N, 05°17′58″E	Grass field	*Arion ater*; *Arion vulgaris*
Bolsøya	62°43′32″N, 07°18′01″E	Strawberry field	*Arion rufus/Arion ater* hybrid; *Arion vulgaris*
Melsomvik	59°15’04″N, 10°21′13″E	Roadside verge	*Arion rufus/Arion ater* hybrid; *Arion vulgaris*
Søgne	58°06′29″N, 07°47′16″E	Grass verge	*Arion fasciatus*
Stavanger	58°58′36″N, 05°44′25″E	Domestic garden	*Arion fuscus*; *Arion vulgaris*

*Type-material.* Holotype female (19 299), paratype female (11 296), paratype male (21 299) and paratype male on a slide with a paratype female (22 294) deposited in USDA (US Department of Agriculture) Nematode Collection, ARS, Nematology Laboratory, Beltsville, MD, USA.

*Site in host*: Isolated from the oesophagus, crop and the buccal mass of slug hosts.

*Representative DNA sequences*: The sequences obtained for *A. norvegicum* n. sp. were deposited in NCBI GenBank under accession numbers KU712560 (SSU rRNA gene), KU712561 (D2D3 LSU rRNA gene) and KU710221 (*cox*1) gene. Sequences of *A. norvegicum* n. sp. were identical across each gene, so only one representative sequence per gene was submitted to GenBank.

*Etymology.* The species name refers to locality of the gastropod host.

### Description (Figs. 1–4)

**Fig. 1:**
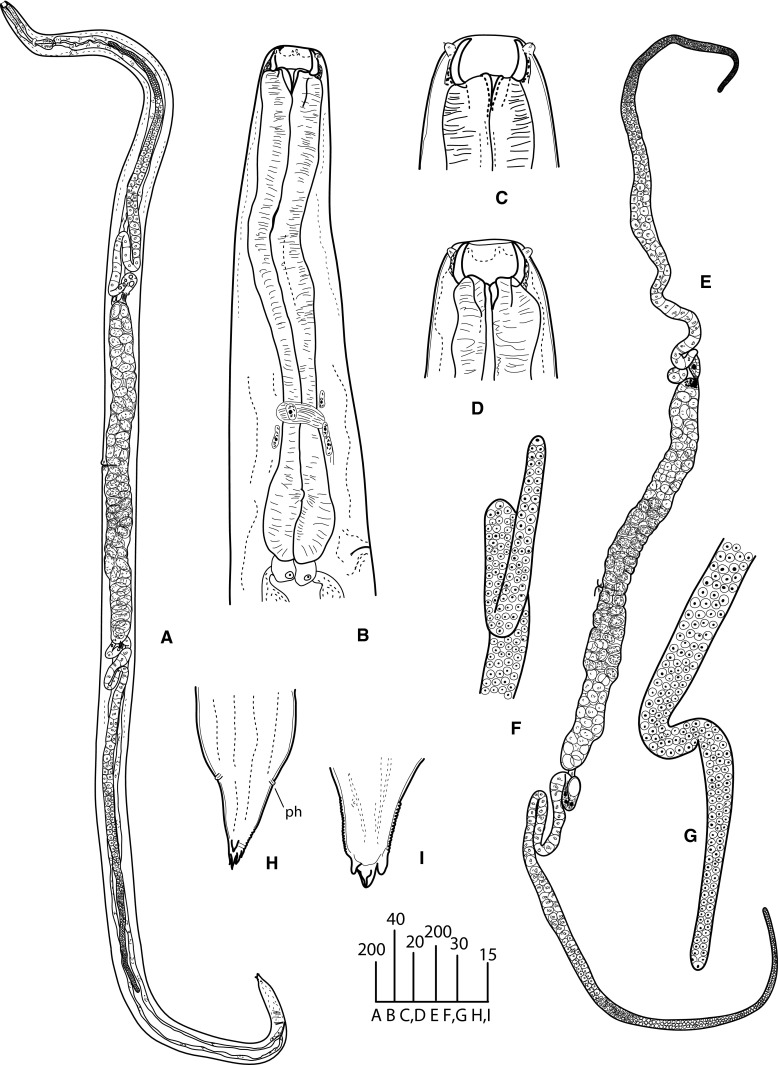
*Angiostoma norvegicum* n. sp. Female. A, Entire worm; B, Anterior region; C, D, Head; E, Gonads; F, G, Distal part of ovaries; H, I, Tail extremity. Except H (ventral), all in lateral view. *Scale-bars* are in micrometres. *Abbreviations*: ph, phasmid

**Fig. 2:**
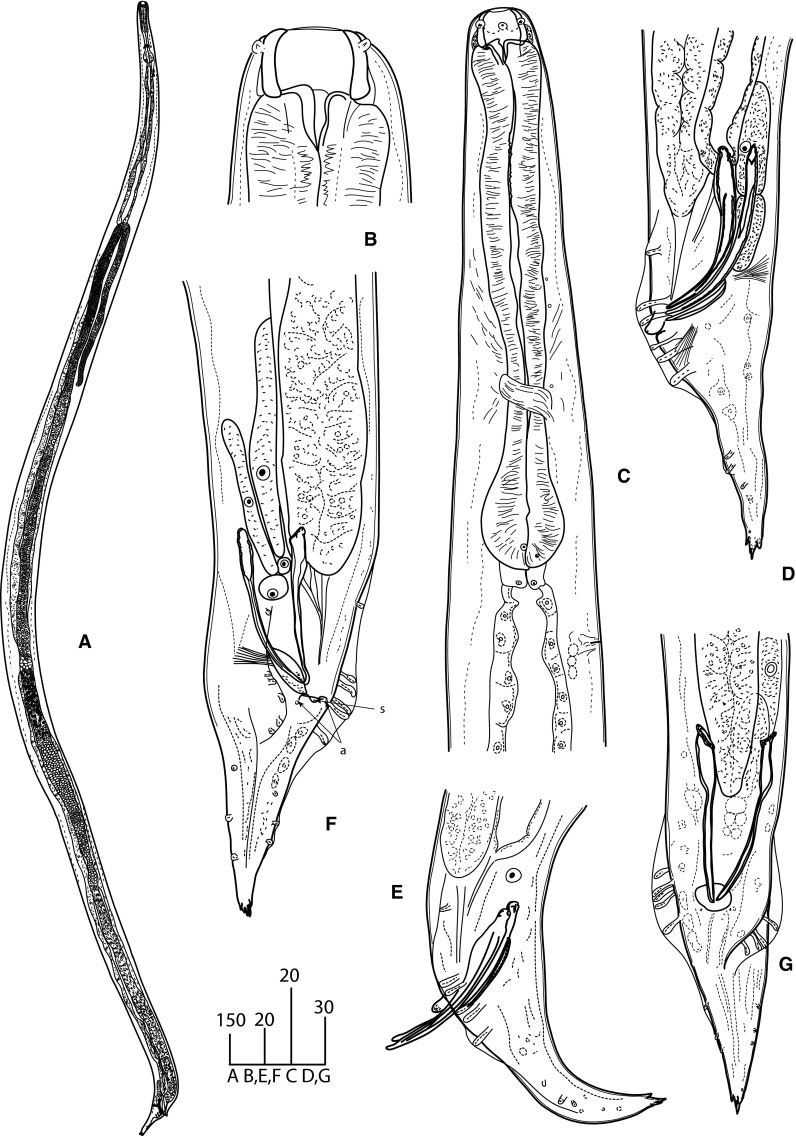
*Angiostoma norvegicum* n. sp. Male. A, Entire worm; B, Head; C, Anterior region; D–G, tail region (F, subventral view; G, ventral view). Except F and G, all in lateral view. *Scale-bars* are in micrometres. *Abbreviations*: s and a, circumcloacal papillae: s, single precloacal papilla; a, anal (postcloacal) papillae

**Fig. 3:**
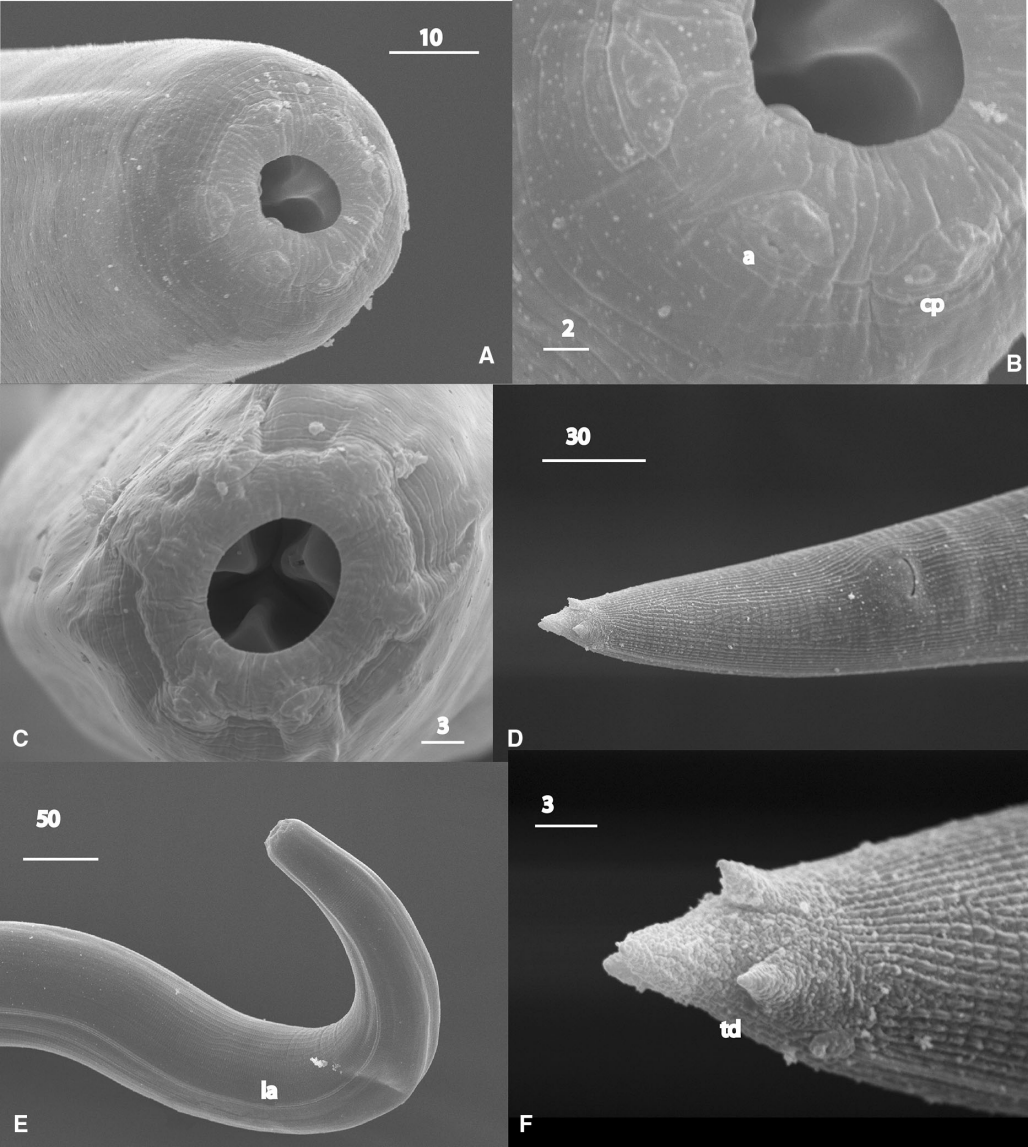
*Angiostoma norvegicum* n. sp. SEM images. A, B, Female head; C, Male head; D, Female tail, ventral view; E, Male anterior region with lateral ala; F, Posterior extremity of female tail. *Scale-bars* are in micrometres. *Abbreviations*: a, amphid; cp, cephalic papilla; la, lateral ala; td, denticle on tail extremity

**Fig. 4:**
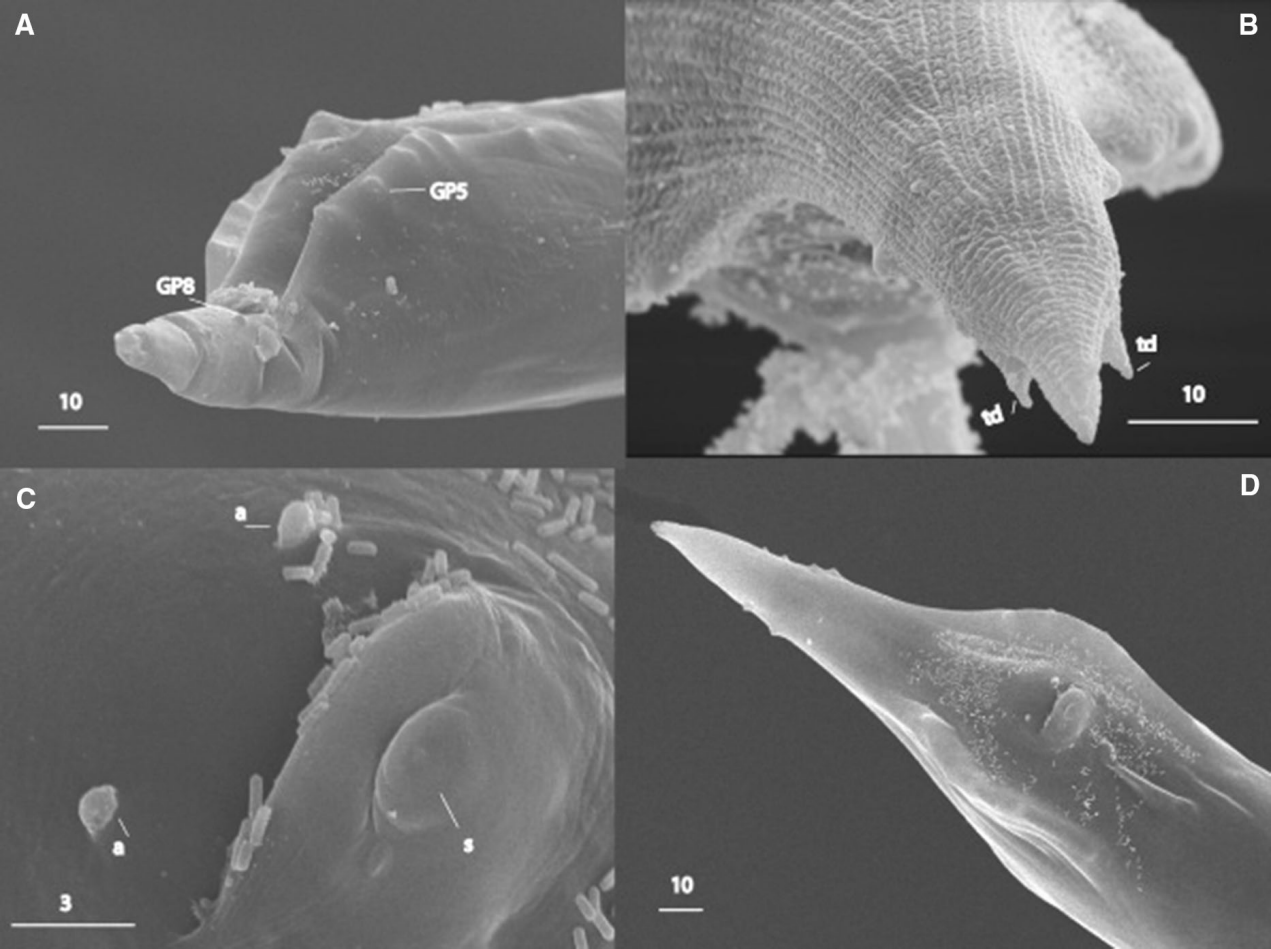
*Angiostoma norvegicum* n. sp. SEM images. Male. A, Tail, sublateral; B, Tail extremity, dorsal view; C, Cloaca; D, Tail, ventral view. *Scale-bars* are in micrometres. *Abbreviations*: GP5 and GP8, genital papillae opened on dorsal side of bursa; s and a, circumcloacal papillae: s, single precloacal papilla; a, anal (postcloacal) papillae; td, denticle on tail extremity

*General*. Body long, cylindrical, slightly tapering to anterior (Fig. 1A, 2A). Cuticle thick, clearly annulated (Fig. 3B). Lateral alae present, starting at mid-pharynx level (Fig. 3E). Head truncated, with 6 salient elevations around mouth aperture, each bearing tiny papilla of internal circle and slightly larger one of external circle except of lateral elevations where pore-like amphids present instead of papillae of external circle (Fig. 3A–C). Mouth aperture wide, circular to broadly oval (Fig. 3A, C). Stoma from 2 parts; anterior part (buccal capsule) spacious, bowl-shaped, with thickened walls 3 (2–5) wide, very slightly concave inside and convex outside; posterior part funnel-shaped, formed by 3 cuticularised, triangular, distally rounded flaps. Posterior funnel-shaped part of stoma *c*.2/3 of buccal capsule length. Externally, bottom half of buccal capsule enveloped by tissue seemingly not representing pharyngeal sleeve (Fig. 1C, D). Pharynx muscular, club-shaped, reaching base of buccal capsule. Pharynx comprises corpus slightly expanded at both ends and occupying 2/3 of total pharynx length, short, narrower isthmus and rounded bulb lacking distinct valves (Fig. 1B). Nerve-ring surrounding mid-isthmus. Excretory pore *c*.1 wide, located near bulb base (Fig. 1B). Cardia prominent. Intestine and rectum well developed, enlarged rectal glands present (Fig. 1A). Tail short, conical, tip supplied with 4 unequal denticles (Fig. 1H, I, 2D–G, 3D, F, 4B).

*Female* [Based on 11 individuals; metrical data in Table 3.] Lateral alae terminating at mid-tail. Buccal capsule 26–34 (30) wide externally, 20–24 (22) wide internally, 14–17 (15) long. Didelphic, amphidelphic. Ovaries distally directed, respectively, to anterior and posterior ends of body (Fig. 1E); both ovaries distally outstretched in *c*.80% of specimens; occasionally, anterior ovary distally forms 1–2 bends keeping the anteriorward direction of top cell; rarely, top cell of anterior ovary directed posteriorward though in this case reflexion of ovary very short (Fig. 1F, G); posterior ovary always outstretched. Top cell of anterior ovary closer to anterior extremity than that of posterior ovary to posterior extremity (mean 569 *vs* 1,067). Proximally, ovaries making two bends followed by short oviducts evidently serving as spermathecae containing large sperm cells *c*.7–8 in diameter. Oviducts leading to spacious, divergent, equally long (*c*.800) uteri. Each uterus containing 40–50 eggs (Fig. 1A). Eggs large, transparent, with smooth, less than 1 thick egg-shells. Oviparous. Vulva slit-like, situated just anterior to mid-body. Vulval lips small. Vagina straight, 40–90 (65) long. Rectum long, with thick lining. Anal lips flat. Phasmids prominent, in 41–50 (45) from tail tip (Fig. 1H).

**Table 3:** Morphometric data for *Angiostoma norvegicum* n. sp. Measurements are in µm and in the form range (mean)

Character	Female		Male
	Holotype	Paratypes	Paratypes
		(n = 10)	(n = 9)
L	8,332	4,540–8,636 (5,946)	4,058–6,476 (5,526)
a	49	28.4–48.3 (41.2)	34.4–51.4 (40.7)
b	26	15.1–29.2 (19.8)	14.5–20.8 (18.6)
c	31.7	22.0–38.5 (30.1)	28.6–44.7 (38.7)
V%	45.2	43.1–49.3 (46)	
Mid-body diameter	170	100–198 (146)	114–169 (137)
Pharynx length	320	264–320 (300)	265–316 (297)
Head to excretory pore	310	206–390 (299)	288–348 (313)
Head to nerve-ring	230	180–230 (206)	180–227 (203)
Tail length	263	134–294 (201)	130–158 (143)
Spicule length (arc)			107–129 (119)
Spicule length (chord)			102–120 (112)
Gubernaculum length			34–48 (42)
Egg length	68	62–72 (66)	
Egg width	40	40–46 (42)	

*Male* [Based on 9 individuals; metrical data in Table 3.] Similar to females in size, body shape and morphology of anterior body end (Fig. 2A, C). Lateral alae terminate at cloaca level. Buccal capsule as long as in females but slightly narrower, 25–29 (27) wide externally, 19–22 (20) wide internally (Fig. 2B). Monorchic. Testis flexure 568–1,052 (810) long, situated at 1/5 body length from anterior extremity (Fig. 2A). Arrangement of spermatocytes and size of spermatids and sperm typical for the genus. Two equal spicules and gubernaculum present. Spicules nearly straight, with small, rounded, poorly separated manubria, proximally expanded shafts, and distal tips rounded, weakly curved. Thin velum present. Gubernaculum plate-like. Pre-anal flap prominent. Circumcloacal papillae 3: one small ventral located on pre-anal flap and 2 larger, pedunculate, subventral, just posterior to lateral margins of cloacal aperture (Fig. 4C, D). Tail broadly conical (Fig. 4D). Bursa rather narrow, not extending to tail tip (Fig. 4D). Genital papillae 9 pairs, pedunculate, incorporated in bursa. Complete formula of genital papillae (GP): 1+2/3+3 with GP 5 and GP 8 opened dorsally. GP1 and GP7-9 short (Fig. 2 D–G, 4A). Phasmids indistinct.

### Remarks

The new species is characterised by its large size and the presence of: lateral alae; a large circular mouth aperture; a spacious stoma; a club-shaped pharynx with a basal bulb without valvular apparatus; a distal part of posterior ovary always outstretched; an anterior ovary distally nearly always outstretched; long, nearly straight spicules and a small gubernaculum; three circumcloacal papillae; caudal genital papillae arranged in a pattern 1+2/3+3 with GP 5 and GP 8 opened on dorsal side of narrow bursa not reaching tail tip; short conical tails in both sexes with tips supplied by 4 short, unequal denticles, and parasitising *Arion* spp. (Table 1).

**Fig. 5:**
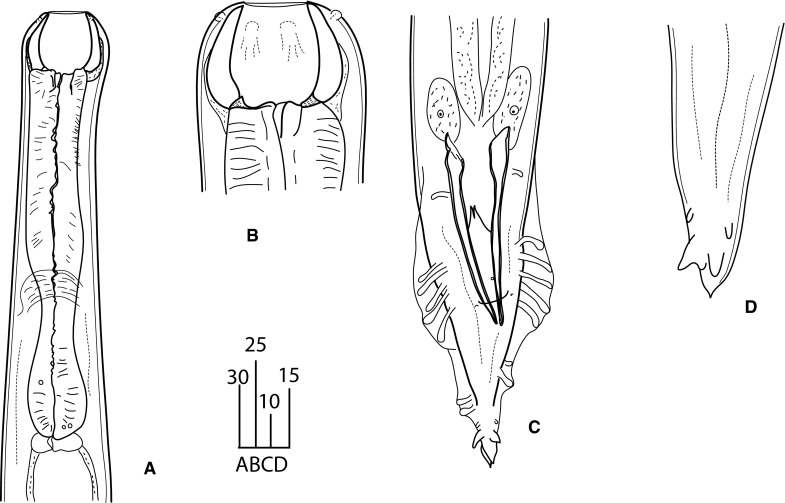
*Angiostoma limacis* Dujardin, 1845 ex *Arion distinctus* Mabille (UK). A, Female, pharynx region; B, Male head; C, Male tail, ventral; D, Female, tip of the tail. Except C, all in lateral view. *Scale-bars* are in micrometres

This new species is morphologically very similar to *A. limacis*, another parasite of the Arionidae, in the body proportions, the shape and structure of the stoma, the shape of the tail in both sexes and the shape and size of spicules and gubernaculum (Mengert, 1953; Morand & Spiridonov, 1989) (Fig. 5C–D). It can be distinguished from the latter by having a shorter stoma (mean 14–17 *vs* 30 µm), the presence (*vs* absence) of lateral alae, the presence (*vs* absence) of a prominent precloacal flap in males, distally outstretched ovaries (always posterior and in majority of cases, also anterior ones) (*vs* reflexed ovaries). Slight differences between these species include thinner stoma walls (mean 3 µm in *A*. *norvegicum* n. sp. *vs* 5 µm in *A. limacis*) and different proportions of stoma (in *A. norvegicum* n. sp., its bowl-shaped portion is shorter while a funnel-shaped one is longer than in *A. limacis*) and pharynx (in *A. norvegicum* n. sp., a procorpus is typically slightly expanded at ends while in *A. limacis*, it is usually uniformly wide with occasional slightest expansion at posterior; also, an isthmus is remarkably much shorter in *A. limacis*, along with the smaller bulb) (Fig. 4A). Also, the head end in the new species is without the slight constriction at the level of stoma base present in *A. limacis* (Fig. 5A–B).

*Angiostoma norvegicum* n. sp. also resembles *Angiostoma asamati* Spiridonov, 1985, *A. milacis* and *A. margaretae*, by having a very similar stoma shape and the presence of lateral alae. However, it can be easily distinguished by the different shape of the tail (short conical with denticles *vs* long conical without denticles) (Spiridonov, 1985; Ivanova & Wilson, 2009; Ross et al., 2011). In the aspect of possessing distally outstretched ovaries, the new species is similar to *A. milacis*, *A. margaretae*, *A. zonitidis* Ivanova & Wilson, 2009 and *A. kimmeriense* Korol & Spiridonov, 1991. From the latter two species, *A. norvegicum* clearly differs by having bowl-shaped stoma with thickened walls (*vs* tubular, thin-walled stoma), not off-set lip region (*vs* off-set lip region), tail tip with denticles (*vs* without denticles), enlarged rectal glands (*vs* normal-sized ones) and different arrangement of genital papillae (Korol & Spiridonov, 1991; Ivanova & Wilson, 2009; Ross et al., 2011).

The tail shape and the presence of lateral alae in the new species represent similarities to *A. aspersae* Morand, 1986. However, *A. norvegicum* differs by lacking a valvular apparatus in the basal bulb and paired papilliform appendages in front of cloaca, and by having denticles on the tail tip, 9 pairs of genital papillae (*vs* 10), longer spicules (mean 119 *vs* 80 µm) and a much larger stoma (Morand, 1986).

## Molecular differentiation and phylogenetic relationships

**Fig. 6:**
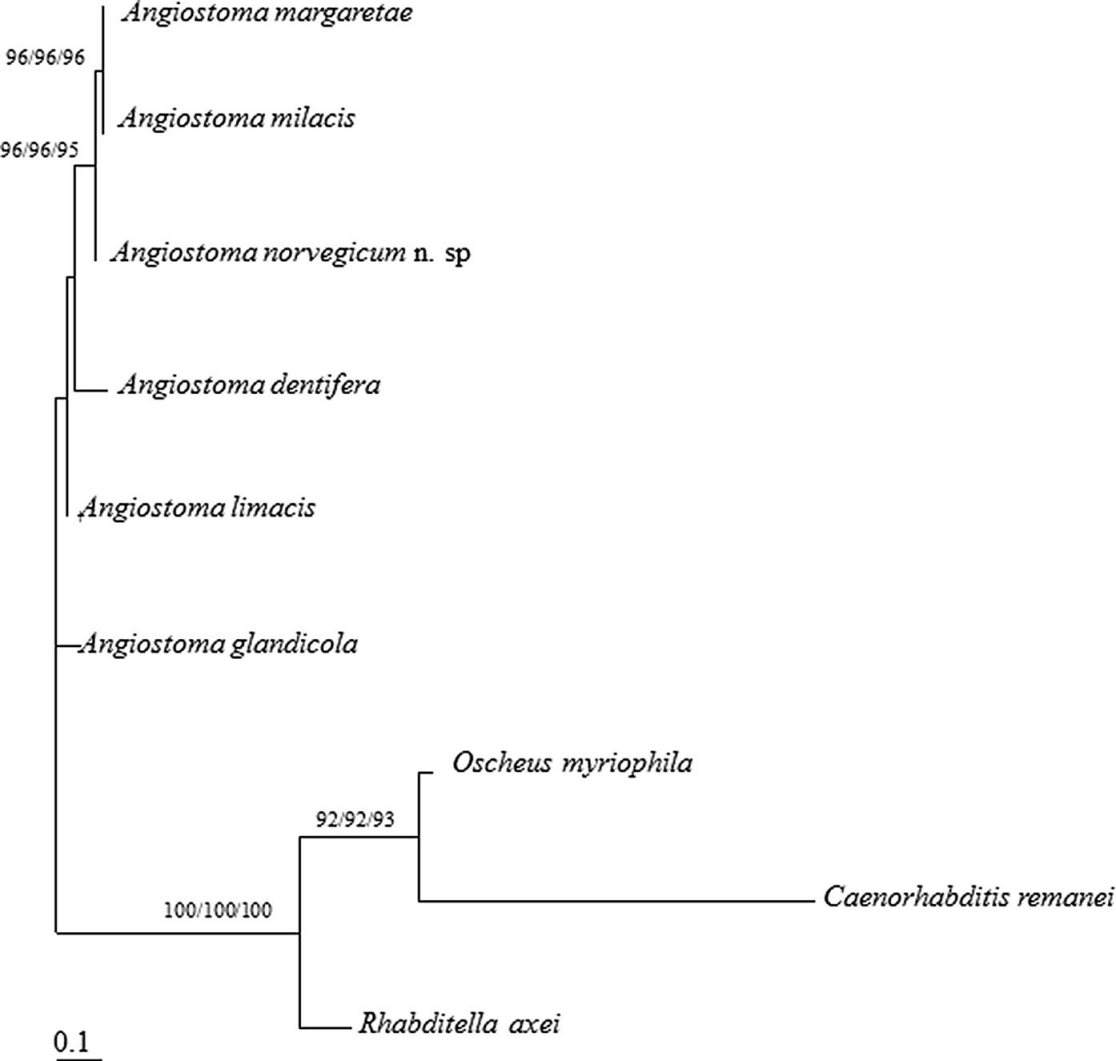
Maximum-likelihood (ML) phylogenetic tree based on D2D3 LSU rRNA gene sequence data for several *Angiostoma* spp. along with selected species of the Rhabditidae used as the outgroup. Phylogenetic analysis of 496 unambiguously aligned nucleotide positions used the GTR correction model with eight gamma-rates and invariable sites. Bootstrap support was calculated based on 1,000 replicates using maximum-likelihood, distance and maximum parsimony methods respectively. Only bootstrap values above 65% are shown

Identical tree topologies were obtained from maximum likelihood (ML), maximum parsimony (MP) and distance analyses. Therefore only the maximum-likelihood tree is presented (Fig. 6) along with bootstrap support from each method of analysis. Phylogenetic analyses demonstrated that the majority of *Angiostoma* spp. cluster together, although only under weak bootstrap support (52/51/53), leaving *A. glandicola* as a sister group to the other *Angiostoma* spp. Within this group, *A. norvegicum* n. sp. formed a monophyletic clade with the two milacid parasites, *A. margaretae* and *A. milacis*, under strong bootstrap support (96/96/95).

## Discussion

The new species, *A. norvegicum* n. sp., is described from five *Arion* slug hosts (*Arion ater*, *A*. *fasciatus*, *A. fuscus*, *A. vulgaris* and *Arion rufus/Arion ater* hybrid) collected throughout Norway. The nematode was isolated from the oesophagus, crop and the buccal mass of its hosts, thus differing somewhat from other *Angiostoma* spp. that have been isolated from the intestine (Mengert, 1953; Spiridonov, 1985; Morand, 1988, 1992; Korol & Spiridonov, 1991; Morand & Barker, 1995), hepatopancreas (Ivanova & Spiridonov, 2010), oesophagus (Ivanova & Wilson, 2009; Ross et al., 2011) and pallial cavity (Morand, 1986) of gastropod hosts.

*Angiostoma norvegicum* n. sp. is extremely prevalent with results similar to that of another arionid-associated species, *A. limacis* from the UK (Ivanova & Wilson, 2009), however mean intensity of infection with *A. norvegicum* n. sp. is higher in all infected host species and particularly in the type-host, *A. vulgaris.* This species also known as the Iberian or Spanish slug, is highly invasive and its current distribution includes mainland Europe, UK, Ireland and USA (Weidema, 2006). However in its native range, which is assumed to be the Iberian Peninsula and the South-West of France, it is much less numerous than in invaded territories (von Proschwitz & Winge, 1994). The species is also known under the name of a ‘killer slug’ because of its habit of feeding on dying and dead slugs. *Arion vulgaris* has the ability to hybridise with the related species of *Arion* thus posing a threat to the native biota (Hatteland et al., 2015). In Norway, the slug was first recorded in 1988 (Hatteland et al., 2013), and is known to inflict serious damage in gardens, horticulture and agriculture.

The limited number of natural enemies of the notorious pest *A. vulgaris* was always considered as one of the traits supporting its success in colonisation of new territories. This study has shown that in Norway *A. vulgaris* does not lack natural enemies (at least regarding nematodes) and even shows a greater susceptibility to slug-associated nematodes than native slug species. This fact can point on the limited ability of the host to tolerate nematode invasion in the conditions of the certain area. The presence of the heavy, combined nematode infection (*A. appendiculatum*, *A. flexilis*, *A. limacis*, *A. norvegicum* n. sp. and *P. hermaphrodita*) of the invasive slug species on the northern boundary of its distribution, together with its absence closer to its native range (Ross et al., 2016), is in opposition to the hypothesis of parasite release, which attributes the success in the establishment of an invader on invaded areas to its release of the natural enemies.

The prevalence of *A. norvegicum* n. sp. in *A. vulgaris* was at the same level as of other larger arionids, i.e*. A. ater* and *A. ater*/*A. rufus* hybrids. The maximum intensity of nematodes per host (56) was the highest ever recorded for a species of the Angiostomatidae. However the absence of *A. norvegicum* n. sp. at certain study sites indicates that the infection of *A. norvegicum* n. sp. was first based on native larger arionids, i.e*. A. ater* and *A. ater*/*A. rufus*. It can be assumed that *A. norvegicum* n. sp. was transmitted to *A. vulgaris* through feeding on other infected *Arion* spp.

Members of the genus *Angiostoma* are not strictly host-specific, however the infection of certain nematode species tends to be based on related host species (Ivanova et al., 2013). In regard to Arionidae, *A. limacis* parasitises arionids and, rarely, agriolimacids of the genus *Deroceras* Rafinesque (see Morand, 1988; Ivanova & Wilson, 2009). In the description of *A. dentiferum*, Mengert (1953) indicated two host species, *Limax cinereoniger* Wolf and *Arion subfuscus* (Draparnaud), from the families Limacidae and Arionidae, respectively. However further studies demonstrated the presence of *A. dentiferum* in *Limax* spp. slugs only (Morand, 1988; Ivanova et al., 2013). Morphologically, *A. dentiferum* is remarkably different from both species of *Angiostoma* parasitising arionid slugs. Thus, *A. norvegicum* n. sp. is the second member of *Angiostoma* associated with slugs of the family Arionidae. Both species, *A. limacis* and *A. norvegicum* n. sp., are very close morphologically. Conversely, the phylogenetic analyses performed in the present study did not support the morphological affinity of these two species. Phylogenetic analyses showed that the majority of *Angiostoma* spp. clusters together, although only under weak bootstrap support, leaving *A. glandicola* as a sister group to the other *Angiostoma* spp. Within this group, *A. norvegicum* n. sp. formed a monophyletic clade with the two milacid parasites, *A. margaretae* and *A. milacis*, thus supporting certain morphological similarities (stoma shape, and the presence of lateral alae, distally outstretched ovaries).

The identification of *A. norvegicum* n. sp. brings the total complement of the genus to 18 species, 14 from terrestrial molluscs and four from amphibian and reptile hosts (Falcón-Ordaz et al., 2008). It has been argued that members of the genus *Angiostoma* are parasites of vertebrates and use molluscs as obligate intermediate hosts (Grewal et al., 2003). However, to date, no *Angiostoma* spp. have been recorded from both invertebrate (supposedly intermediate) and vertebrate (definitive) hosts. In addition, *Angiostoma* spp. parasitic in both molluscan and vertebrate hosts have been isolated in their adult stages, indicating that they are in their final phase of development. The resolution of the genus can only be confirmed through molecular and phylogenetic analysis of the genus; however, to date insufficient molecular data are available for *Angiostoma* spp. from vertebrate hosts.
